# Fluctuating sea-level and reversing Monsoon winds drive Holocene lagoon infill in Southeast Asia

**DOI:** 10.1038/s41598-023-31976-z

**Published:** 2023-03-28

**Authors:** Yannis Kappelmann, Hildegard Westphal, Dominik Kneer, Henry C. Wu, André Wizemann, Jamaluddin Jompa, Thomas Mann

**Affiliations:** 1 Geoecology and Carbonate Sedimentology Group, Leibniz Centre for Tropical Marine Research (ZMT), Bremen, Germany; 2 Department of Geosciences, University of Bremen, Bremen, Germany; 3 Physical Science and Engineering Division (PSE), King Abdullah University of Science and Technology (KAUST), Thuwal, Kingdom of Saudi Arabia; 4 Bioplan GmbH, Ostseebad Nienhagen, Germany; 5 Research and Development Center for Marine, Coastal, and Small Islands, Hasanuddin University, Makassar, Indonesia; 6 Federal Institute for Geosciences and Natural Resources, Hannover, Germany

**Keywords:** Climate sciences, Ecology, Environmental sciences

## Abstract

Many lagoons surrounded by reefs are partially or completely infilled with reef-derived detrital carbonate sediment. Sediment deposits in such restricted environments are archives of prevailing environmental conditions during lagoon infill. For Indonesia, no paleoenvironmental reconstructions based on Holocene lagoon sediments exist. Here we analyze the sedimentary record obtained from five percussion cores penetrating 10 m into the unconsolidated subsurface of a reef island in the Spermonde Archipelago, Indonesia. The combined compositional, textural and chronostratigraphic analyses reveal that the sedimentary infill of the lagoon underlying the island, starting 6900 years cal BP, was interrupted between 5800 and 4400 years cal BP, when sea level was ~ 0.5 m higher than at present, and monsoon intensity was lower. After the intensity of the monsoons increased to modern levels, and sea level dropped to its present position, lagoonal sedimentation was re-initiated and created the foundation for an island that built up since 3000 years cal BP. Our study provides the first geological evidence for the strong sensitivity of detrital carbonate systems in Indonesia to fluctuations in sea level and dominant wind direction. It thus sheds light on how changing environmental conditions in the context of global warming could affect the morphological development of reef systems, and thereby also habitable coastal areas.

## Introduction

Characterized by reefs that encircle and shelter a deeper interior, lagoons provide accommodation space for sediment, in many cases forming shallow, planar zones in their final stages of infilling^1,2^⁠⁠. The sedimentary lagoon infill may originate in-situ from biogenic production in the lagoon or may be transported to the lagoon from surrounding reefs^3^⁠. This leads to different types of infilling, also on regional scale, where for example some lagoons in the Maldives are predominantly infilled by in-situ sedimentation^1,4^⁠. Other Maldivian lagoons^5^ and some lagoons from the Great Barrier Reef^6^⁠ and the Mascarenes^7^⁠, however, receive most of their sediment through transport from adjacent reefs into the lagoon.

Lagoon development is controlled by the interplay between the growth and productivity of the biological carbonate secreting reef ecosystem, sea-level change and the water movement above adjacent reefs, which affects both, accommodation space and sediment transport^2,8,9^⁠. Sea-level lowstands can cut off lagoons temporarily from sediment supply^2,6^⁠ while sea-level highstands typically promote infill through an increase in the hydrodynamic energy across the reef platforms^10,11^⁠.

**Figure 1:**
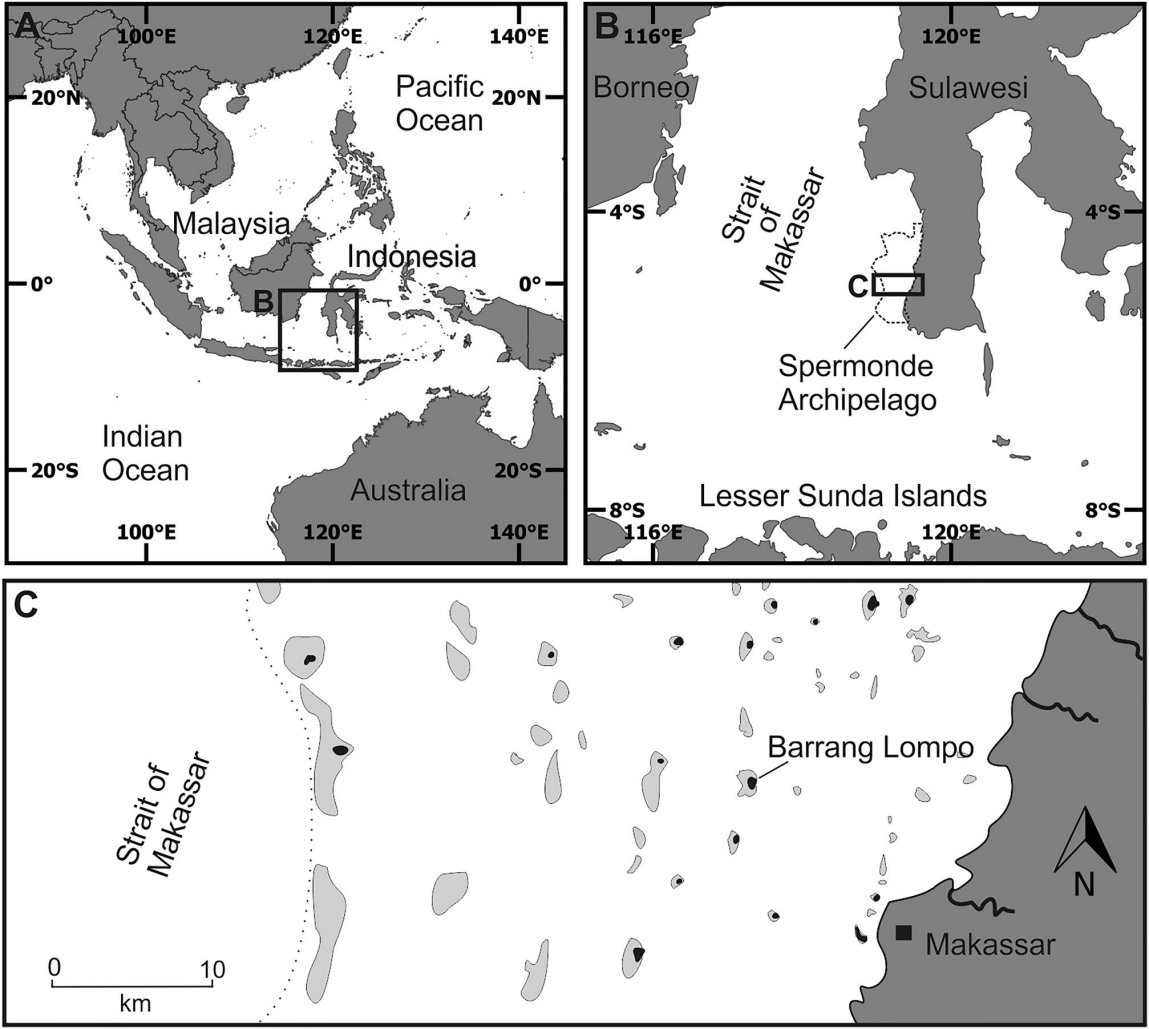
The location of studied island in the Spermonde Archipelago. (**A**) The Indonesian Archipelago in SE Asia; (**B**) the Spermonde Archipelago located between SW Sulawesi and the Strait of Makassar; (**C**) Barrang Lompo island on the mid-shelf, light grey structures mark larger submerged reefs, black areas represent islands (following Kench and Mann^12^).

For the present study of a Holocene lagoon infill and subsequent reef-island formation in the Spermonde Archipelago, Indonesia (Fig. 1), five sediment cores were studied. The sediment cores were 10 m deep each, with sampling intervals of 1 m, providing a total of 50 samples for grain size and skeletal component analyses. To gain insights into the depositional mode (i.e. autochthonous vs. allochthonous) and the ecological background through time, we analyzed the carbonate facies supported by radiocarbon dating of picked and pooled benthic foraminifera.

## Study area

**Figure 2:**
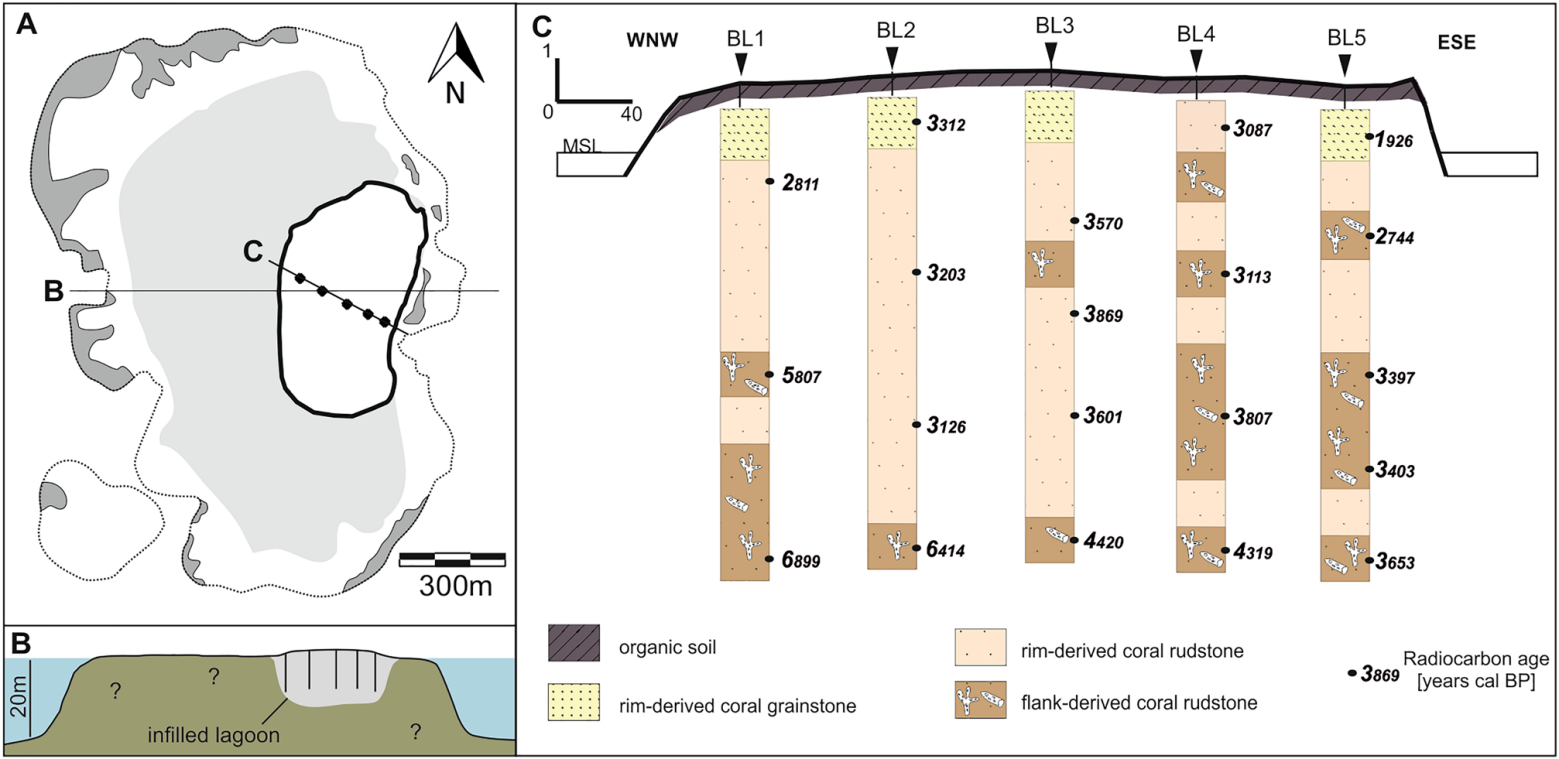
(**A**) Barrang Lompo patch reef, zonation follows Sawayama et al.^19^: dark grey—reef flat with living coral, white—reef flat with coral rubble, light grey—sand cover on reef flat, bold black line—island; (**B**) W-E profile of the patch reef, based on handheld echo sounder data; (**C**) spatial distribution of facies obtained from cores BL1 to BL5 and calibrated radiocarbon ages from 20 samples.

The broad and shallow carbonate shelf of the Spermonde Archipelago, offshore SW Sulawesi, Indonesia (Fig. 1) is characterized by reef morphologies ranging from a barrier reef on the margin in the west, adjacent to the Strait of Makassar, to numerous patch reefs throughout the shelf which have deeper lagoons or form shallow reef flats and often host low-lying reef islands^12^⁠. The mid-shelf island studied here, Barrang Lompo (Fig. 1), is one of the largest islands in the Spermonde Archipelago with 4600 inhabitants over an area of 21.2 ha^13^⁠. It is located in the east of a patch reef complex (Fig. 2A) that emerges from water depths of around 20 m (Fig. 2B). Most of the inner reef flat is characterized by a sandy layer, ranging from few decimeters to ~ 1 m thickness, covering a layer of large coral blocks. This solid layer can be found close to the borehole BL1, a few meters from the coastline towards northwest (Fig. 2B).

Reversing monsoon winds are the main driver of wave and sediment dynamics in the area, with a dominant NW monsoon during boreal winter and a weaker SE monsoon in boreal summer^12,14^⁠. Contrary to these contemporary conditions, the early Holocene wind regime was characterized by a stronger SE monsoon, the intensity of which declined until the mid-Holocene. In parallel the intensity of the NW monsoon increased over the past 4000 years^15,16^⁠. Sea-level reconstructions based on measurements of fossil microatolls from five different islands indicate that the Spermonde Archipelago had a higher relative mean sea-level (henceforth referred to as relative sea-level or RSL) in the mid-Holocene. The area experienced a highstand of ~ 0.5 m from around 5700 years cal BP from which it fell to the contemporary level until around 4200 years cal BP^17,18^⁠.

## Results

**Figure 3:**
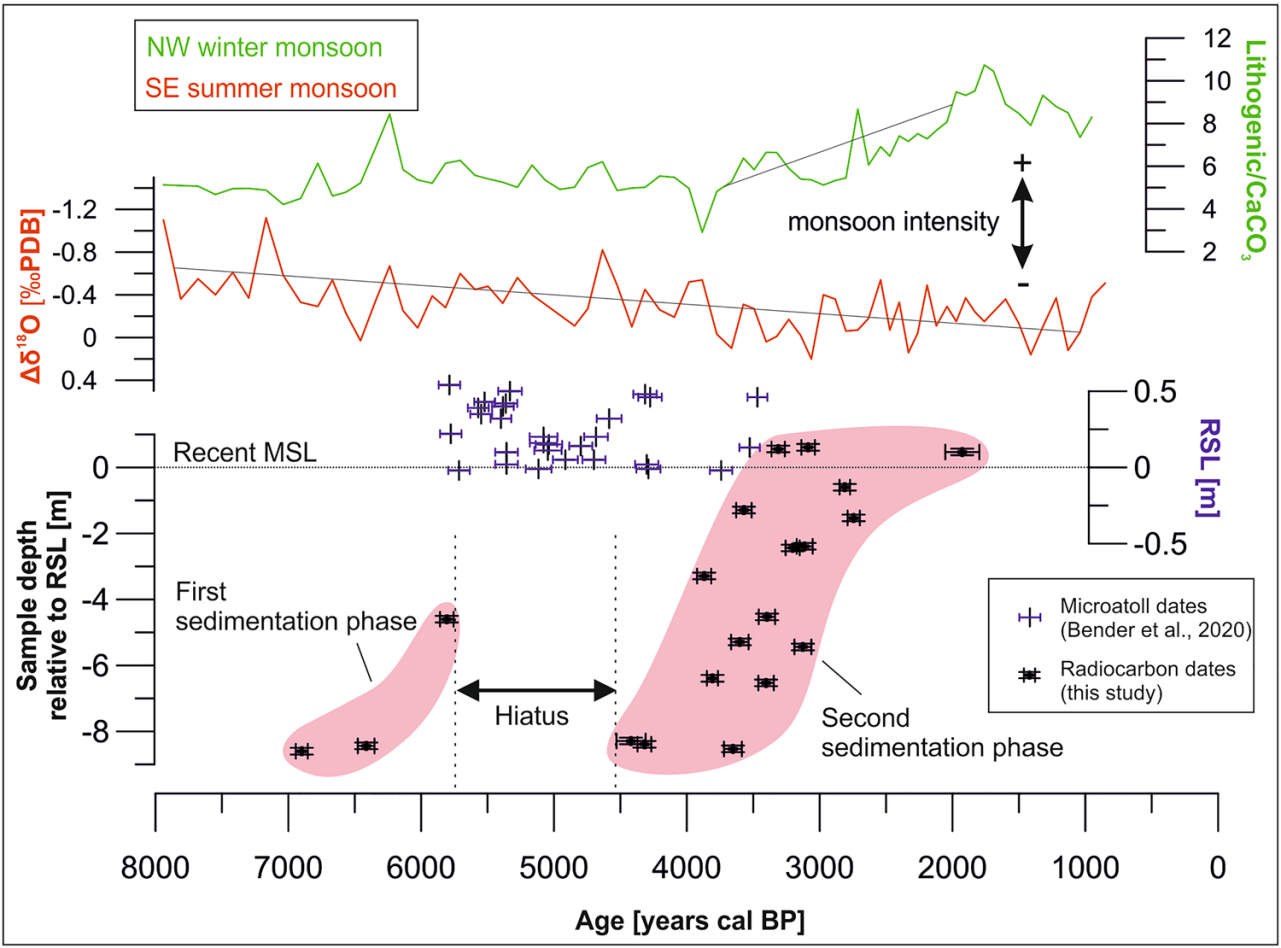
Two phases of sedimentation (light red polygons) in the context of rising sea-level and monsoon intensities. Calibrated ages from the present study (black points), plotted relative to RSL, including horizontal error bars for age error. Monsoon data are based on sediment core GeoB 10,053–7 from offshore Java in Mohtadi et al.^16^, available in the PANGAEA data repository (https://doi.org/10.1594/PANGAEA.767353). The SE monsoon (red) weakens throughout the Holocene, the NW monsoon (green) intensifies from 4000 years BP onwards. The proxy for the intensity of the SE monsoon is based on the ratio of Δ^18^O in two planktonic foraminifera, of which one is indicative for upwelling intensity and thus monsoon intensity. The NW monsoon proxy is based on the ratio of lithogenic (terrestrial) material and biogenic CaCO_3_, of which higher rates of the former indicate stronger monsoon intensity and run-off (for a full description of parameters see original source^16^). Sea-level data report a highstand from around 5700 years cal BP onwards (data from Bender et al.^18^, available in the PANGAEA data repository under https://doi.org/10.1594/PANGAEA.917685), coinciding with the hiatus.

The sediments recovered from the cores are composed of sandy to gravelly, unconsolidated, reef-derived carbonate components, dominated by coral fragments and gastropods (~ 60% and ~ 20%, respectively; see Supplementary Tables ST1, ST2 and ST3). Minor constituents include bivalves, foraminifera, and *Halimeda*. Sand-sized components commonly show abrasion, whereas larger coral fragments in pristine condition mainly occur in samples from deep core sections. Three distinct subsurface facies occur: rim-derived coral grainstone, rim-derived coral rudstone and flank-derived coral rudstone (see Supplementary Table ST4). The calibrated radiocarbon ages suggest that the studied complex formed between 6900 and 1900 years cal BP during two phases (sedimentation phases SP1 and SP2; Fig. 2 and 3, and Supplementary Table ST5). SP1 covers the timeframe from 6900 to 5800 years cal BP and is represented by flank-derived coral rudstone facies that incorporates large pristine coral fragments. Sediments of SP1 are exclusively found in the deep core sections in the NW part of the lagoon (cores BL1 and BL2; Fig. 2). Between 5800 and 4400 years cal BP no sedimentation is recorded in our cores (Fig. 3). SP2 sets in from 4400 to 1900 years cal BP, when the deposition of rim-derived coral rudstone is predominant especially in the NW cores, while the cores in the SE contain both rim- and flank-derived coral rudstone. The majority of radiocarbon dates in SP2 reveal ages between 3800 and 3200 years cal BP, younger ages are recorded in relatively deep core sections in the NW (e.g. core BL2, 7 m depth: 3126 years cal BP; see Fig. 2). The few minor (< 300 years) age inversions in individual cores were likely caused by the mixing of foraminifera during transport, so that we regard this a robust chronostratigraphic framework. The late stages of SP2 until 1900 years cal BP include shallow lagoon and island sediments, the latter of which are mainly composed of the finer rim-derived coral grainstone.

## Modes of lagoon infill

In all cores, the skeletal composition of the sediments in the lagoon is largely similar. This implies that the sediment source, the supplying reef system, did not experience major shifts throughout the process of infill. With coral fragments being dominant components, and gastropods being common, the sedimentary facies indicates a shallow reef or reef flat as sediment source^6,20,21^⁠. The abraded condition of most components supports that a large part of the sediment was exposed to reworking prior to deposition in the lagoon^7,22^. At the same time, the absence of indicators of autochthonous lagoonal sedimentation, such as fine-grained sediment and *Halimeda* plates (see Supplementary Table ST3) differs from sediment infill in other lagoons in the Indo-Pacific, where in-situ sedimentation of *Halimeda* is a major constituent^1,4,23^. We thus interpret the facies of the Barrang Lompo lagoon to indicate that sediment production on surrounding reefs and the subsequent transport into the lagoon were the main drivers for lagoon infilling. The spatial distribution, the presence of coarse, flank-derived rudstone facies in the most NW and SE locations and the absence of this facies in the central core indicate that sediment was supplied into the lagoon from the marginal reefs on both sides. The pristine coral fragments in the flank-derived sediments are interpreted to stem from proximal reefs on both sides, from which they were transported and deposited quickly, rather than being exposed to abrasive processes for a longer time^7,23^⁠.

The transition from flank-derived coral rudstone to the overlying rim-derived coral rudstone marks a significant facies shift through a decrease in mean grain size, a stronger abrasion of components and the absence of pristine coral fragments. The higher degree of abrasion and smaller mean grain sizes of the rim-derived sediments implies that they were exposed to more effective physical alteration processes prior to deposition^24,25^⁠. Extended plain surfaces of reef flats are known to act as temporary sediment storage where sediment is reworked and fragmented prior to deposition, and they act as an effective pathway to the lagoon as shown by previous studies e.g. in New Caledonia^26^⁠ and the Great Barrier Reef^27^⁠. Thus, the facies transition suggests that the contemporary reef flat prograded towards NW and provided material for lagoon infill. This is supported by the age reversals in SP2, since they likely indicate that the analyzed foraminifera tests remained in an environment where they were mixed with other tests of different ages prior to transport into the lagoon. Furthermore, this would also explain the absence of pristine coral fragments in the rim-derived facies, since sedimentation would lead to burial and die-off of coral^7,28^⁠. Our data therefore suggest that the extensive reef flat formed shortly after sea level had fallen to the contemporary level around 4200 years cal BP, which coincides with observations from other Indo-Pacific reefs which have been shown to catch up and develop reef flats around 4000 years BP^17,18,20,29^⁠.

## Mid-Holocene hiatus

The radiocarbon ages suggest that the lagoon infill was interrupted between 5800 and 4400 years cal BP when no sedimentation is recorded in our cores (Fig. 3). A relative sea-level highstand of ~ 0.5 m from 5700 on to around 4200 years cal BP coincides with this hiatus^17,18^⁠. This likely explains the onset of the hiatus and is in contrast to observations of increased rates of lagoon infill under rising sea-level^5,30^⁠. Sedimentation after the hiatus in the southeastern cores is recorded from 4400 years cal BP, and the temporal gap in the northwestern cores lasted until 3200 years cal BP. This makes it unlikely that the subsequent sea-level fall has immediately triggered the continuation of lagoon infill as seen elsewhere^11,26^. Hence, the shifting monsoon winds probably influenced lagoon infill during the Holocene, as they are known to significantly affect reef morphology and sediment dynamics in the study area^12,14,31^⁠. Since the early Holocene, the formerly strong SE monsoon steadily decreased in intensity and was replaced as dominant wind system by the NW monsoon around 6000 years BP. Due to orbital forcing and increased insolation in the northern hemisphere, the intensity of the latter increased from 4000 years BP onwards, leading to contemporary conditions (Fig. 3)^15,16,32^. These dynamics are more broadly related to changing climate in the Indo-Pacific region^33^, the global effects of which extend to the high latitudes^34,35^. For the present study, it is likely that the prevailing direction of sediment transport evolved according to the new dominant wind direction. The reef flat located in the NW of the recent island, or former lagoon, was probably formed around 4000 years cal BP and acted as sediment source. We hypothesize that the reversal in dominant monsoon wind direction favored the transport of rim-derived sediments into the lagoon, thus completing the infill, and soon thereafter initiating island formation. Our observations are therefore consistent with other studies of reef evolution that have described the profound impact of a changing climate in the mid-Holocene^36,37^. In addition, other climate effects may have contributed to the observed sea-level variations. The South China Sea, the main contributor of low salinity water for the Indonesian Throughflow in the Strait of Makassar^38^, experienced pronounced fluctuations in salinity during the mid-Holocene^33^. Variability in salinity and sea surface temperature in the Strait of Makassar during the Holocene^39^ could have caused steric effects adding to sea-level fluctuations^17,18^ influencing island formation in the study area.

## Infill model and island formation

**Figure 4:**
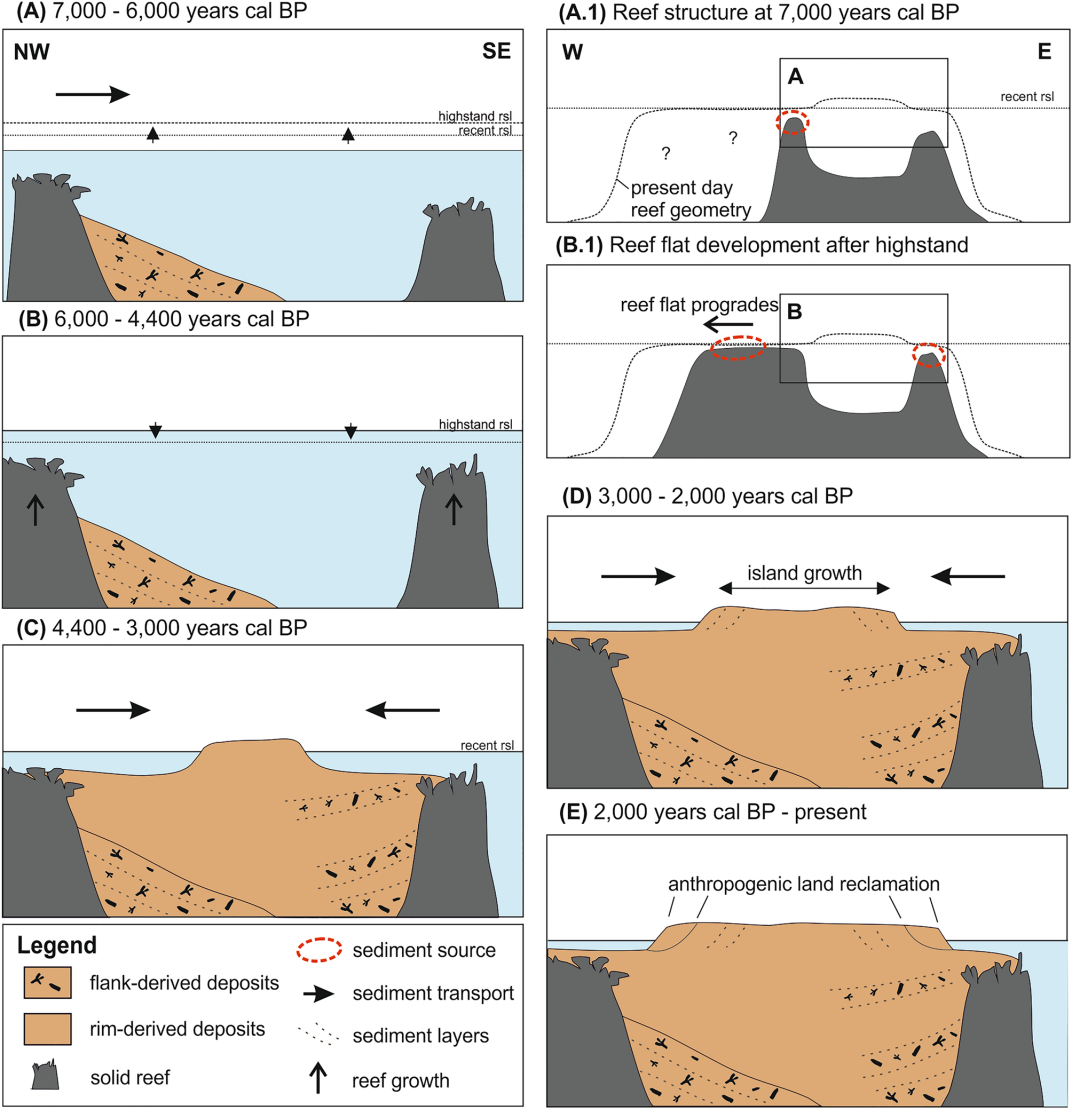
Evolutionary model over the past 7000 years. (**A**) Initial sedimentation in NW under rising sea-level, derived from proximal reef flanks with (**A.1**) individual reefs developed; (**B**) hiatus during highstand and subsequent sea-level fall, allowing reefs to catch-up and (**B.1**) to develop flat-topped reef through progradation; (**C**) infill of the lagoon, promoted by finer, rim-derived material in NW and flank-derived material in SE with subsequent formation of island nucleus; (**D**) completion of infill and simultaneous island growth; (**E**) stability and land reclamation on the flanks.

Based on our findings we conceptualize a lagoon infill and island formation model, in which three distinct, local sediment sources were dominant: a proximal reef in the NW and a proximal reef in the SE, which provided flank-derived carbonate facies, and the shallow reef flat in the NW of the lagoon or island that provided rim-derived sediment (Fig. 4). Initial sedimentation during SP1 is recorded from 7000 to 5800 years cal BP (Fig. 4A), when flank-derived sediments prograded from the proximal reef in the NW into the lagoon. Sea-level was rising and the SE monsoon was dominant during this first phase of sedimentation^15–18^⁠. We found no record of sedimentation in the following 1400 years in the lagoon (Fig. 4B), and no further lateral progradation in the NW is recorded. This hiatus coincides with the time when sea-level reached a highstand of 0.5 m and intensity of the SE monsoon steadily decreased. From 4400 years cal BP lagoon infill sedimentation increased in the SE (SP2), mainly through deposition of flank-derived rudstone sourced from a proximal reef in the SE (Fig. 4C). In the NW, the hiatus lasted until around 3200 years cal BP until accumulation of rim-derived facies re-initiated sedimentation. This material originated from the reef flat in the NW of the lagoon that had developed after sea level had fallen to the contemporary level by 4200 years cal BP. While the onset of sedimentation in the SE may be connected to falling sea-level, the delayed input of sediment from the reef flat in the northwest may be linked to intensifying monsoon winds towards northwest from 4000 years BP (Fig. 3), facilitating increased transport into the lagoon. The infill was completed around 3000 years cal BP and soon thereafter, the initial island developed. Until 2000 years cal BP, the island accreted towards NW and SE (Fig. 4D). At latest in the seventeenth century, humans settled on the island^40^ (Fig. 4E).

## Conclusion

Our results indicate that the studied lagoon in the Spermonde Archipelago was infilled by the transport of sediment from the surrounding reefs. Importantly, our chronostratigraphic data suggest that the process of infill was influenced and slowed down by a rise in sea level, but we detected no clear dependence between the subsequent sea-level fall and re-initiation, and later completion of the lagoon infill. The reversal in dominant monsoon wind direction thus likely played a key role in lagoon infill and island formation. This underlines the sensitivity of regional reef islands to changes in both, sea level and wind patterns.

## Material and methods

### Field methods

Five locations were cored across a SE-NW transect with a direct push drill rig (percussion drilling). The rig consisted of a jackhammer with an internal combustion engine (WACKER BH 23, Wacker Neuson SE), connected to a direct push corer with liner of 46 mm diameter (Nordmeyer Geotool GmbH). Samples were retrieved manually with a “Zweistangen-Handziehgerät 60 kN” and liners were then removed from the corer and capped for transport to the laboratory. A total of n = 50 samples were obtained for analysis. For each location, maximum depth was 10 m below island surface. Samples were taken from the lower sections of the probes in order to avoid contamination of samples. From each meter, one sample from 10 cm probe-length was obtained. Due to compression of ~ 50%, samples were assumed to mirror intervals of 20 cm. Data on location and surface height were obtained using GPS during field work and are shown in Table 1.

**Table 1:** Five core locations on Barrang Lompo with elevation of the core location and maximum drilling depth, each with respect to mean sea-level.

Location	Latitude	Longitude	Height above RSL	Maximum depth sampled relative to RSL
[]	[°]	[°]	[m]	[m]
BL1	5°2′52.5″S	119°19′40.1″E	1.30	− 8.70
BL2	5°2′53.7″S	119°19′42.3″E	1.46	− 8.54
BL3	5°2′54.9″S	119°19′44.6″E	1.61	− 8.39
BL4	5°2′55.9″S	119°19′46.6″E	1.51	− 8.49
BL5	5°2′56.6″S	119°19′48.2″E	1.37	− 8.63

### Grain size

Samples were oven-dried at 40 °C for 72 h in the laboratory. Dried samples were divided into working halves and archive halves, using a rifle splitter. Working halves were dry-sieved into fractions following^41^⁠, ranging from − 4 to + 4 Φ (see Supplementary Table ST1). Grain size statistics were evaluated using GRADISTAT v4.0^42^ with implemented formulas of Folk and Ward^43^ (see Supplementary Table ST2).

### Component analysis

Skeletal components were determined using a digital microscope. From each sample, a total of 500 skeletal grains were identified. Unidentifiable grains were counted as bioclasts, yet not included in the sum of 500. From the factions <  − 1 Φ (Gravel) and up to 0 Φ (Very Coarse Sand) 200 components were identified. From the fraction up to 1 Φ (Coarse Sand) 100 components were identified (see Supplementary Table ST3). The number of counted components was reduced in the coarse sand fraction and smaller fractions were left out in general, due to high degree of abrasion and respective high numbers of unidentifiable grains. In case fractions did not provide the targeted number of identified components (200 and 100, respectively), the next smaller fraction was used to compensate the deficit.

### Facies description

Grain size parameters and proportions of skeletal analysis were used to group sediments and find descriptive terms for different facies. Nomenclature is inspired by initial suggestions by Dunham^44^ and Wright^45^⁠, and respective application for soft carbonate sediments e.g. in Perry et al.^1^⁠ and Gischler et al.^46^⁠. Samples comprising < 10% gravelly material (> 2 mm) are termed “grainstone”, whereas samples with > 10% gravelly material are termed “rudstone”. Furthermore, the dominating component has been considered in the classification (e.g. coral-dominated grainstone). Lastly, the occurrence (yes/no) of fresh coral fragments > 16 mm was chosen as indicator for the sediment source (e.g. flank-derived or rim-derived), assuming that the abundant occurrence of non-abraded Acropora sp. thickets reflect a proximal sediment source (see Supplementary Table ST4).

### Radiocarbon analysis

The foraminifera *Calcarina* spp. was picked for determination of radiocarbon ages from 20 samples, since it was present in all samples (compared to other common dating material, such as fresh coral sticks or *Halimeda*). To minimize the lag time between the death of the organism and the deposition of the skeletal remains, the most pristine tests (i.e. tests with best preserved spines and surface) were picked. Cumulative mass of at least 20 mg foraminifera tests were picked from each sample, the number of picked specimens per sample ranged from n = 13 to n = 24. Each specimen was classified in terms of abrasion following Fellowes et al.^26^⁠ (see Supplementary Table ST6). Dating was undertaken at the Poznan Radiocarbon Laboratory.

### Calibration

Conventional radiocarbon ages were calibrated using the MARINE20 Marine Radiocarbon Age Calibration Curve^47^ implemented in the online version OxCal v4.4^48^⁠ (see Supplementary Table ST5). The regional marine reservoir correction used was ΔR = 0 ± 0. This value is given as an approximation (“close to zero”) in Southon et al.^49^, based on unpublished data from the Strait of Makassar. Although a value for the regional marine reservoir correction in this area is reported in Southon et al.^49^ (South Borneo, ΔR = 74 ± 70), the mentioned value close to zero was chosen. As argued by Bender et al.^18^, given the distance of more than 900 km between the sampling site in South Borneo^49^ and the Spermonde Archipelago, and the intervening shielding Strait of Makassar, the regional marine reservoir correction of ΔR = 0 ± 0 was applied. The unit *years cal BP* is used for the (re)calibrated radiocarbon ages^17,18,50^ from the study area with ΔR = 0 ± 0.

## Supplementary Information

Supplementary Information 1.

Supplementary Information 2.

## Data Availability

The datasets used and analyzed during the current study are available from the PANGAEA data repository^50^.
