# Supplementary material for: Fluctuating sea-level and reversing Monsoon winds drive Holocene lagoon infill in Southeast Asia
Source: Sci Rep. 2023 Mar 28;13:5042. doi: 10.1038/s41598-023-31976-z (PMC10050433; doi:10.1038/s41598-023-31976-z)
Supplement: Supplementary file 2 — Supplementary Information 2. [file 41598_2023_31976_MOESM2_ESM.pdf]

S3_Supp_Images

Image data of picked and pooled foraminifera of the genus Calcarina spp. for AMS radiocarbon dating.

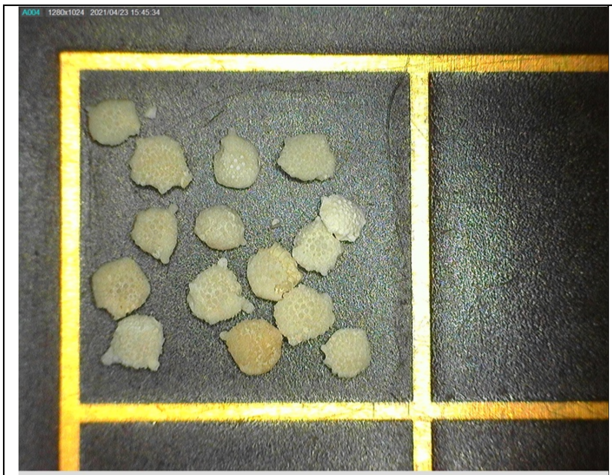	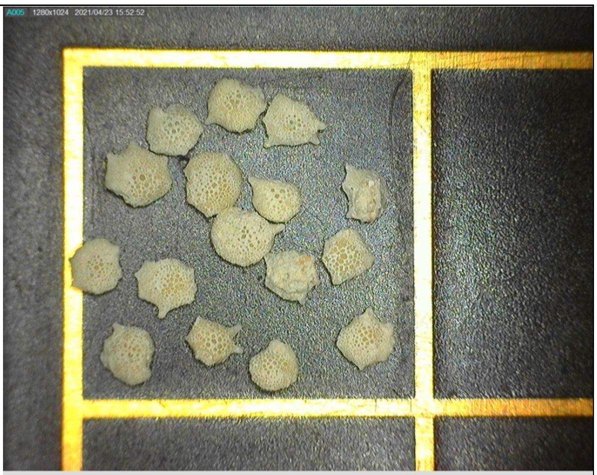
BL1-2	BL1-6
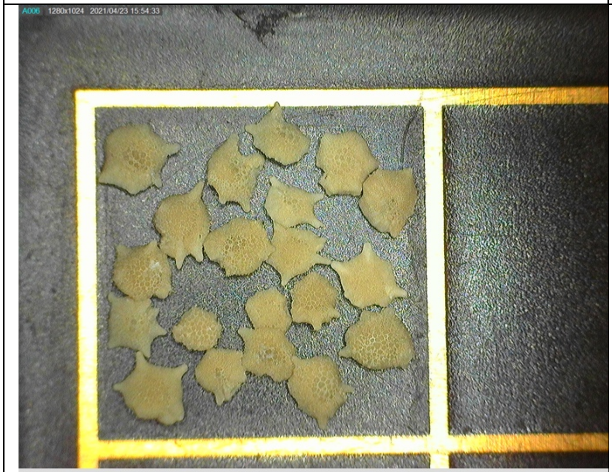	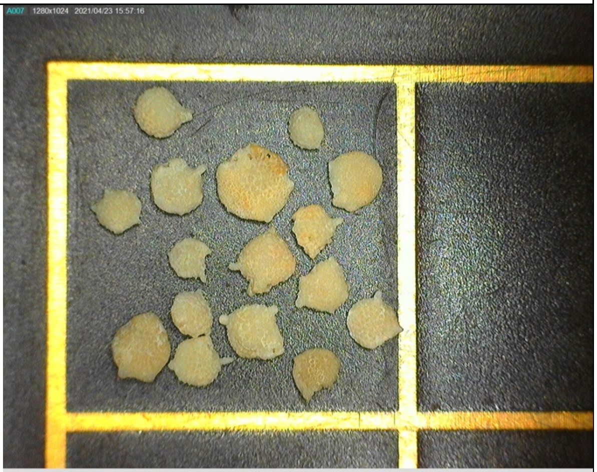
BL1-10	BL2-1
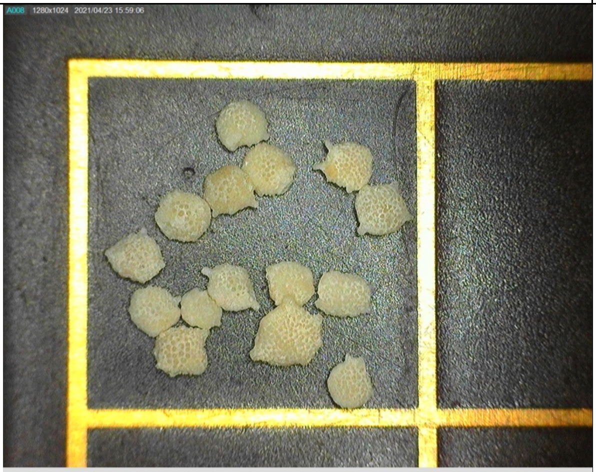	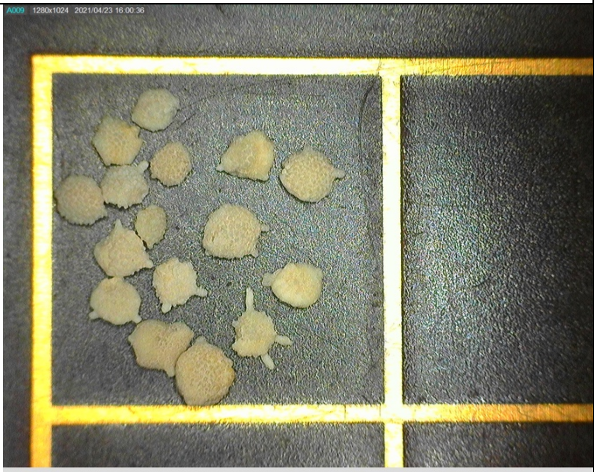
BL2-4	BL2-7

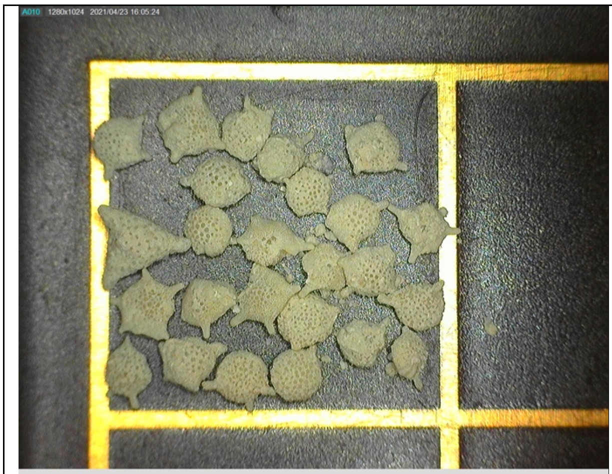

BL2-10

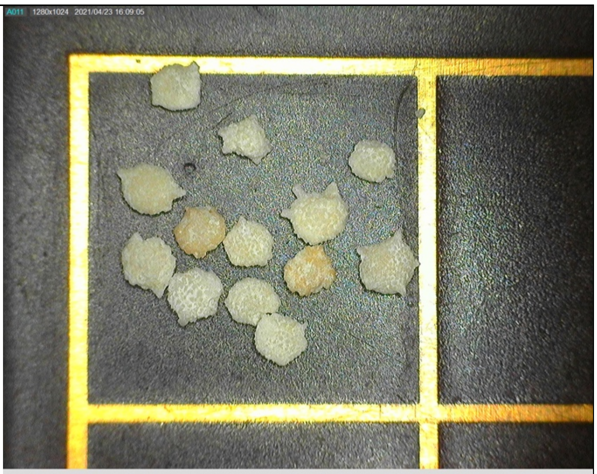

BL3-3

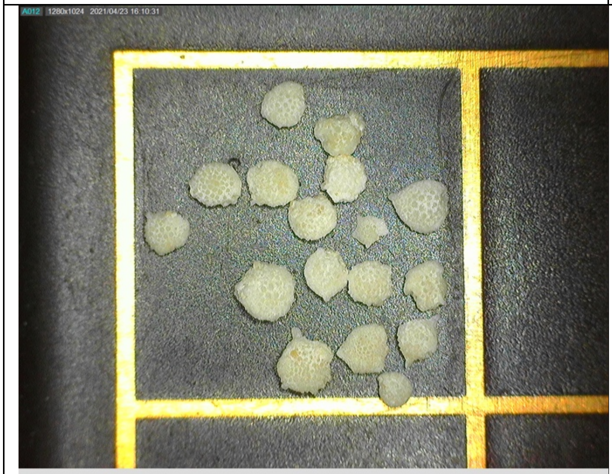

BL3-5

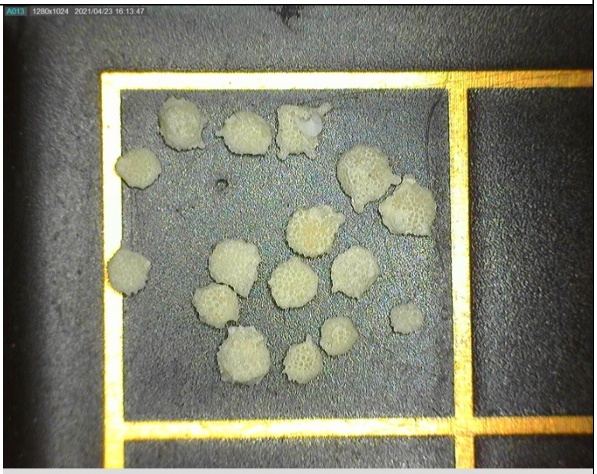

BL3-7

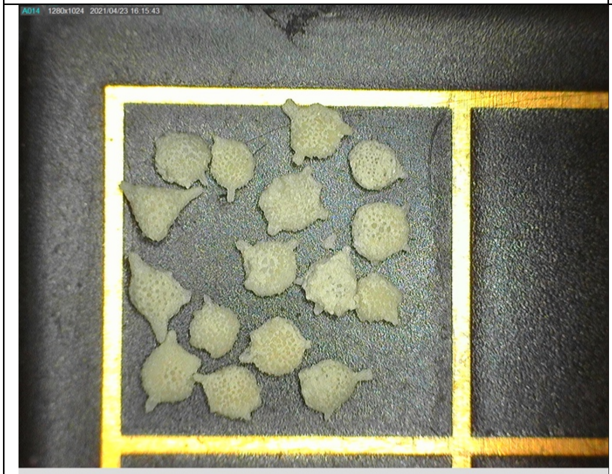

BL3-10

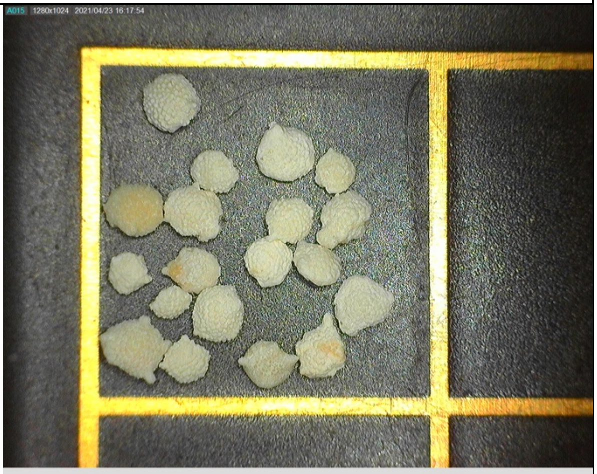

BL4-1

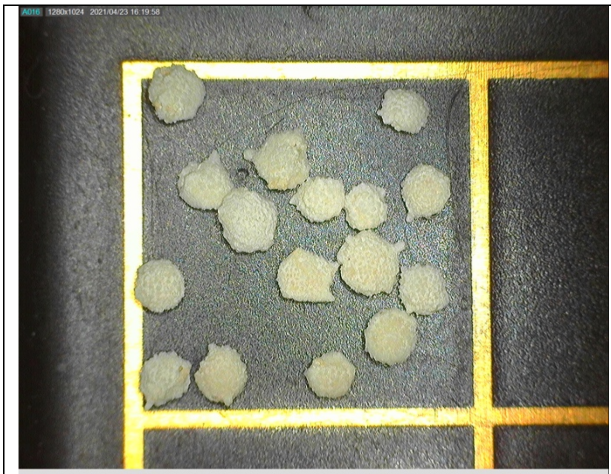

BL4-4

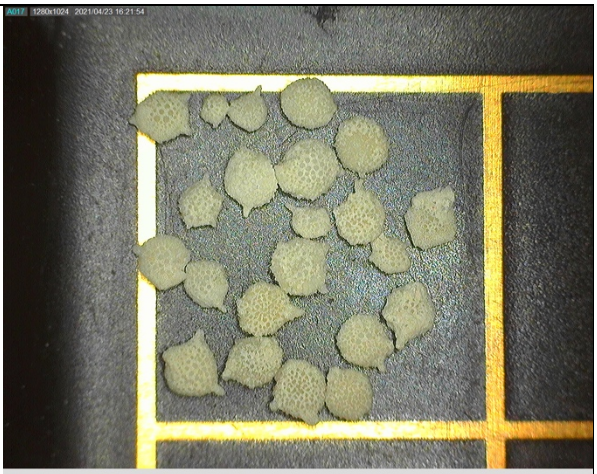

BL4-8

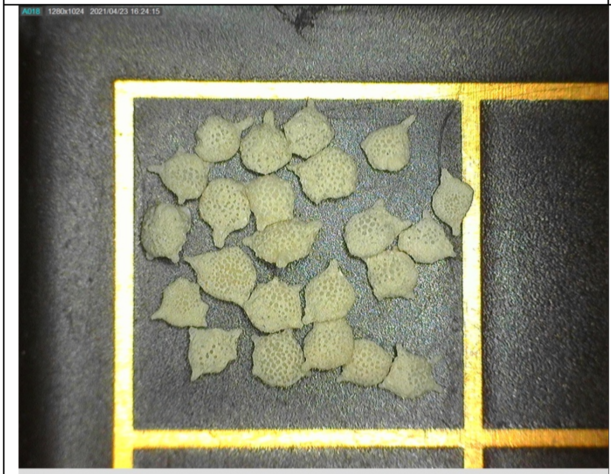

BL4-10

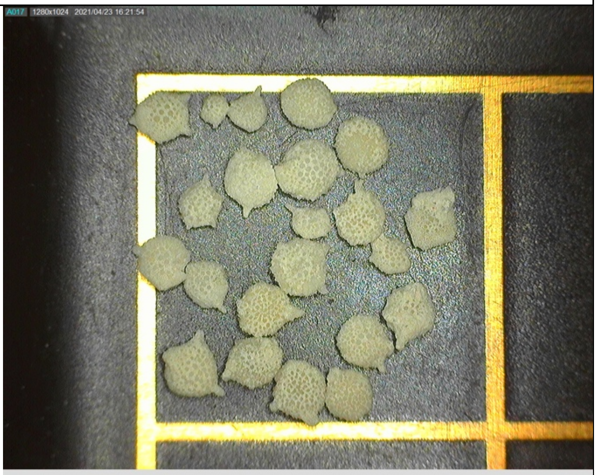

BL5-1

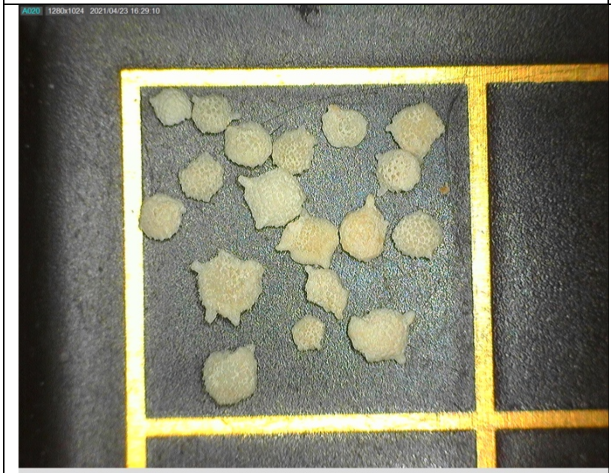

BL5-3

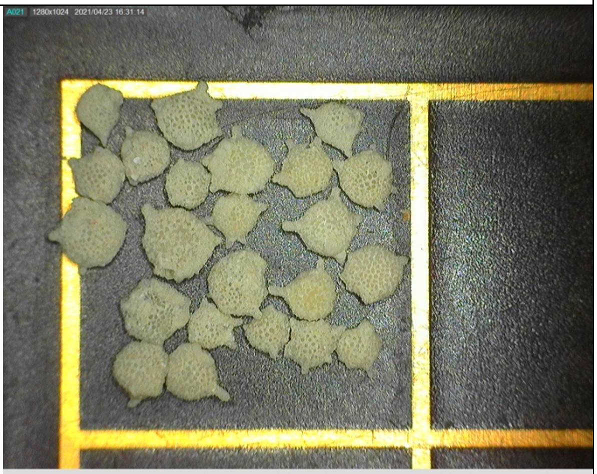

BL5-6

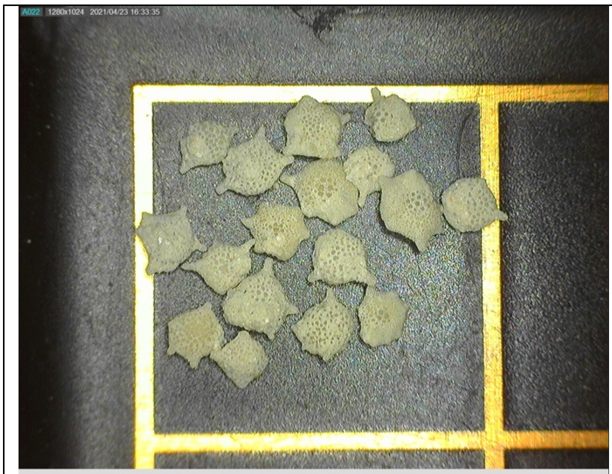	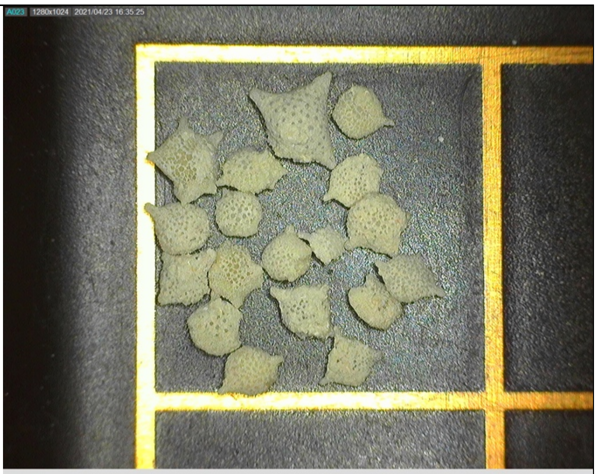
BL5-8	BL5-10
